# Lipid Peroxidation-Derived Aldehydes, 4-Hydroxynonenal and Malondialdehyde in Aging-Related Disorders

**DOI:** 10.3390/antiox7080102

**Published:** 2018-07-30

**Authors:** Giuseppina Barrera, Stefania Pizzimenti, Martina Daga, Chiara Dianzani, Alessia Arcaro, Giovanni Paolo Cetrangolo, Giulio Giordano, Marie Angele Cucci, Maria Graf, Fabrizio Gentile

**Affiliations:** 1 Dipartimento di Scienze Cliniche e Biologiche, Università di Torino, 10124 Turin, Italy; stefania.pizzimenti@unito.it (S.P.); martina.daga@unito.it (M.D.); marieangele.cucci@unito.it (M.A.C.); 2 Dipartimento di Scienze e Tecnologia del Farmaco, Università di Torino, 10124 Turin, Italy; chiara.dianzani@unito.it; 3 Dipartimento di Medicina e Scienze della Salute “V. Tiberio”, Università del Molise, 86100 Campobasso, Italy; alessia.arcaro@unimol.it (A.A.); gianpaolo.cet@tiscali.it (G.P.C.); gentilefabrizio@unimol.it (F.G.); 4 Presidio Ospedaliero “A. Cardarelli”, Azienda Sanitaria Regione Molise, 86100 Campobasso, Italy; giuliogiordano@hotmail.com (G.G.); mariagraf@tiscali.it (M.G.)

**Keywords:** aldehydes, osteopenia, sarcopenia, myelodysplastic syndromes, immunosenescence

## Abstract

Among the various mechanisms involved in aging, it was proposed long ago that a prominent role is played by oxidative stress. A major way by which the latter can provoke structural damage to biological macromolecules, such as DNA, lipids, and proteins, is by fueling the peroxidation of membrane lipids, leading to the production of several reactive aldehydes. Lipid peroxidation-derived aldehydes can not only modify biological macromolecules, by forming covalent electrophilic addition products with them, but also act as second messengers of oxidative stress, having relatively extended lifespans. Their effects might be further enhanced with aging, as their concentrations in cells and biological fluids increase with age. Since the involvement and the role of lipid peroxidation-derived aldehydes, particularly of 4-hydroxynonenal (HNE), in neurodegenerations, inflammation, and cancer, has been discussed in several excellent recent reviews, in the present one we focus on the involvement of reactive aldehydes in other age-related disorders: osteopenia, sarcopenia, immunosenescence and myelodysplastic syndromes. In these aging-related disorders, characterized by increases of oxidative stress, both HNE and malondialdehyde (MDA) play important pathogenic roles. These aldehydes, and HNE in particular, can form adducts with circulating or cellular proteins of critical functional importance, such as the proteins involved in apoptosis in muscle cells, thus leading to their functional decay and acceleration of their molecular turnover and functionality. We suggest that a major fraction of the toxic effects observed in age-related disorders could depend on the formation of aldehyde-protein adducts. New redox proteomic approaches, pinpointing the modifications of distinct cell proteins by the aldehydes generated in the course of oxidative stress, should be extended to these age-associated disorders, to pave the way to targeted therapeutic strategies, aiming to alleviate the burden of morbidity and mortality associated with these disturbances.

## 1. Introduction

In recent years, increased life expectancy due to an improved quality of life and decline in mortality rates, is leading to a society in which the aging population is growing more rapidly than the entire population. Life expectancy is projected to increase in industrialized countries. In 2016, 27.3 million very old adults were living in the European Union, and in the UK, 2.4% of the population (1.6 million) were aged 85 and over [1]. It has been calculated that there is more than a 50% probability that, by 2030, female life expectancy will exceed the 90-year barrier, a level that was deemed unattainable at the turn of the 21st century [2]. The functional disturbances appearing in old age are referred to as the “aging process”, which entails changes in body composition, imbalances in energy production and use, homeostatic dysregulation, neurodegeneration and loss of neuroplasticity [3]. The scientific community has formulated over 300 theories to explain the driving forces behind aging [4], but none has proven, so far, to be universally applicable. The free radical theory of aging has gained widespread acceptance, thus becoming one of the leading explanations of the aging process at the molecular level. It is commonly believed that the aging process is related to an imbalance favoring pro-oxidant over antioxidant molecules and a consequent increase of oxidative stress [5]. Oxidative stress entails elevated intracellular levels of reactive oxygen species (ROS), which can cause damage to proteins, lipids, and DNA. The mitochondria are the primary sources of intracellular ROS (~1–5%), due to the electron leakage primarily resulting from the electron transport chain [6]. Indeed, mitochondrial dysfunction has long been considered a major contributor to aging and age-related diseases. In elderly subjects, mitochondria are characterized by functional impairment, such as lowered oxidative capacity, reduced oxidative phosphorylation, decreased adenosine triphosphate production, significantly increased ROS generation, and diminished antioxidant defense [7]. Depending on their concentration in the cells, ROS are either physiological signals essential for cell life or toxic species which damage cell structure and functions. In particular, ROS cause the oxidation of polyunsaturated fatty acids in membrane lipid bilayers, leading eventually to the formation of aldehydes, which have been considered as toxic messengers of oxidative stress, able to propagate and amplify oxidative injury [8].

**Figure 1:**
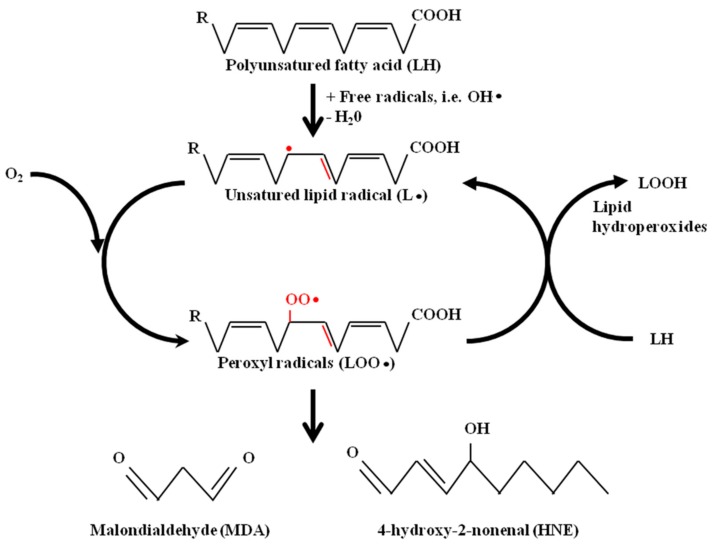
Malondialdehyde (MDA) and 4-hydroxynonenal (HNE) formation from polyunsaturated fatty acids.

Among the aldehydes produced by lipid peroxidation (LPO), malonaldehyde (MDA) and 4-hydroxynonenal (HNE) have gained most attention, since MDA is produced at high levels during LPO, so that it is commonly used as a measure of oxidative stress, and HNE has been shown to be endowed with the highest biological activity. The production, metabolism, and signaling mechanisms of two main omega-6 fatty acids lipid peroxidation products, MDA and HNE, have been extensively studied and reported in an excellent review [9]. Briefly, MDA and HNE originate from the peroxidation of polyunsaturated fatty acids. LPO can be described generally as a process under which oxidant, free radical or non-radical chemical species attack lipids containing carbon–carbon double bond(s). This process involves hydrogen abstraction from a carbon atom and oxygen insertion, resulting in the formation of peroxyl radicals and lipid hydroperoxides, as illustrated in Figure 1.

Since aldehydes have high chemical reactivities, mammals have evolved a full set of enzymes converting them into less reactive chemical species and contributing to the control of their intracellular concentrations, which reflect the steady-state between the rates of formation by LPO and catabolism into less reactive compounds. Once formed, MDA and HNE can be reduced to alcohols by aldo-keto reductases or alcohol dehydrogenases, or can be oxidized to acids by aldehyde dehydrogenases [10]. Moreover, HNE, which is the most reactive among aldehydes, easily reacts with low-molecular-weight compounds, such as glutathione. This reaction can occur spontaneously or can be catalyzed by glutathione-S-transferases [10]. HNE and MDA are able to affect several signaling processes. Most of these effects depend on their ability to bind covalently to proteins and DNA.

### Aldehyde–Protein Adduct in Human Disease

LPO-derived aldehydes easily react with proteins, generating a wide variety of intra- and inter-molecular covalent adducts. Depending on their structural features, they can form Schiff bases and/or Michael adducts with the free amine group of lysine, the imidazoline group of histidine, the guanidine group of arginine and the thiol group of cysteine [11]. HNE can affect cell functions, through its ability to form adducts with proteins involved in signal transduction and gene expression, including receptors, kinases, phosphatases and transcription factors [11]. MDA can form protein adducts by specifically modifying the lysyl residues of proteins [12], and modified autologous biomolecules can generate neoepitopes from self epitopes, capable of inducing undesired innate and adaptive immune responses, including atherosclerosis [13]. HNE adducts can accumulate progressively in the vascular system, leading to cellular dysfunctions and tissue damaging effects, which are involved in the progression of atherosclerosis. Moreover, HNE, by forming HNE-apoB adducts, contributes to the atherogenicity of oxidized low-density lipoproteins (LDL), leading to the formation of foam cells [14]. The presence of HNE-protein adducts has been detected in inflammation-related diseases, such as alcoholic liver disorders and chronic alcoholic pancreatitis, in which the increased formation of HNE-protein adducts was evidenced in acinar cells adjacent to the interlobular connective tissue [15]. Moreover, HNE-protein adducts have been detected in brain tissues and body fluids in several neurodegenerative diseases, such as Alzheimer’s disease, Huntington disease, Parkinson’s disease, amyotrophic lateral sclerosis, and Down syndrome [10].

The involvement of MDA and HNE in age-related diseases has been supported by the observations that the concentrations of MDA and HNE, in human erythrocytes and blood plasma, increased during the aging process [16]. The role of reactive aldehydes in age-related pathologies, such as atherosclerosis [13], neurodegenerations [17], inflammation, and cancer [10] has been discussed by many excellent reviews. Other age-related disorders, such as osteopenia/osteoporosis, sarcopenia, immunosenescence and myelodysplastic syndromes have received less attention. In this review, we focus our attention on to recent advances in the role of LPO-derived aldehydes and aldehyde-protein adducts in these age-related disorders, all of which are characterized by increases of oxidative stress and consequent increases in the generation of LPO-derived aldehydes.

## 2. Osteopenia/Osteoporosis

Bone remodeling is a highly dynamic physiological process: osteoblasts (bone-forming cells) and osteoclasts (bone-resorbing cells) work simultaneously to maintain bone density and strength [18]. During aging, bone density decreases and the measure of bone mineral density is commonly employed to evaluate whether or not a patient is affected by osteopenia or osteoporosis. Osteopenia is the thinning of bone mass. Such a decrease in bone mass is not usually “severe”, but is considered a very serious risk factor for the development of osteoporosis. Osteoporosis is characterized by profound losses of skeletal mass, coupled with architectural deterioration, which increase bone fragility and susceptibility to fractures. Several recent papers have provided insights into the current prevalence of osteoporosis in specific older populations. The current National Osteoporosis Foundation guidelines for the USA, coupled with the most recently available population data from the National Health and Nutrition Survey, show that the eligibility for osteoporosis treatment increases exponentially with age; roughly 10% of both men and women meet criteria for treatment at age 50 years, whereas 48% of men and 79% of women over 80 years meet treatment guidelines [19]. Current treatments for osteoporosis, which increase bone density and reduce fracture risk, include anti-resorptive medications (bisphosphonates and denosumab), which primarily increase endocortical bone and cortical thickness, and anabolic medications (teriparatide and abaloparatide), which increase the periosteal and endosteal perimeters, without causing large changes in cortical thickness [20].

Increases of ROS levels have been observed consistently in osteopenia and osteoporosis. In aging people, the number and activity of individual osteoblasts decrease and osteoblast apoptosis increases, in association with oxidative stress-induced osteoporosis [21]. Some studies explored the relationships between the use of antioxidants and bone metabolism. Indeed, a marked decrease in plasma antioxidants was found in aged or osteoporotic rats and in aged or osteoporotic women [22,23,24]. The loss of antioxidant capacity leads to accelerated bone loss through the activation of a tumor necrosis factor alpha (TNF-α)-dependent signalling pathway [25]. In turn, the administration of antioxidants such as vitamin C, E, N-acetyl-cysteine and linoleic acid, had beneficial effects in individuals with osteoporosis [26].

Osteoblast apoptosis was induced by oxidative stress through the activation of the c-Jun N-terminal kinase (JNK) pathway [27] and the NF-kB pathway [28]. In unstimulated cells, NF-κB proteins are sequestered in the cytoplasm because of their association with IκB (inhibitor of κ light gene enhancer in B cells) proteins. Phosphorylation and degradation of IκB disrupt this association and allow the translocation of NF-κB proteins into the nucleus. ROS induce the oxidation of critical cysteines and enhance the activity of several cytoplasmic kinases, which promote IκB phosphorylation and degradation, including IκB kinase and the PKC family of serine/threonine kinases [29]. Additionally, ROS-induced modifications control key steps in the nuclear phase of the NF-κB program, including recruitment of coactivators, chromatin remodeling, and DNA binding. The activation of NF-kB in osteoblastic cells increased the phosphorylation of p66 (shc) protein, which amplified mitochondrial ROS generation and stimulated apoptosis [29]. The increase of ROS in osteoblastic MC3T3-E1 cells induced phosphorylation of MAPKs (mitogen-activated protein kinases), which subsequently triggered the intrinsic apoptosis pathway. Moreover, the advanced oxidation protein products induced osteoblast apoptosis in aged Sprague–Dawley rats [30].

The NF-κB pathway is involved in osteoclast activity, as well. Indeed, ROS stimulated osteoclast differentiation and activity through activation of the NF-κB pathway, and these effects were reversed upon NF-κB suppression [31].

Other than by the direct actions mentioned above, ROS can affect bone turnover by enhancing the production of LPO-derived reactive intermediates, thereby causing protein damage and inflammatory responses [32]. In vitro experiments have proved that HNE, the most biologically active aldehyde produced by LPO, can induce intense oxidative stress, inflammatory reactions, and apoptosis in osteoblasts, via the induction of protein phosphatase 2A activity, which has earned a reputation as a mediator of oxidative stress-induced apoptosis in these cells [21]. The role of LPO-derived aldehydes in osteoblast apoptosis is also supported by studies on aldehyde dehydrogenases (ALDH), a family of enzymes involved in aldehyde degradation [33]. ALDH inhibition by disulfiram resulted in bone loss in rats [34]. Additional confirmation of the role of ALDH activity in osteoporosis comes from the results obtained with transgenic mice expressing the Aldh2*2 (Aldh2*2 Tg) dominant-negative form of ALDH2. These transgenic mice exhibited severe osteoporosis, indicating that ALDH2 regulates physiological bone homeostasis. Moreover, the Aldh2*2 transgene or treatment with acetaldehyde induced the accumulation of HNE and the expression of peroxysome proliferator-activated receptor γ (PPAR-γ) a transcription factor that promotes adipogenesis and inhibits osteoblastogenesis [35]. On the contrary, the activation of ALDH2 by *N*-(1,3-benzodioxol-5-ylmethyl)-2,6-dichlorobenzamide (alda-1) had an osteogenic effect, involving increased production of bone morphogenetic protein-2 by osteoblasts [36]. Taken together, these experimental data seem to delineate a role for the products of LPO in the induction of osteoporosis. However, the measurements of MDA as an indicator of LPO in post-menopausal osteoporotic women gave conflicting results. One study disclosed significant increases in plasma MDA concentrations in post-menopausal osteoporotic women, compared with control subjects [37], whereas another one failed to detect changes of MDA concentrations between osteoporotic and non-osteoporotic post-menopausal women [38]. Thus, further studies are needed to precisely define the role played by LPO-derived aldehydes and to validate the possible inclusion of targeted therapies aimed to reducing the effects of reactive aldehydes in the development and progression of age-related osteopenia/osteoporosis.

## 3. Sarcopenia

Sarcopenia is a geriatric syndrome characterized by a progressive and generalized loss of skeletal muscle mass, together with low muscle strength and/or poor physical performance in the elderly [39]. A recent study in Brazilians aged 60 years or older demonstrated that the overall prevalence of sarcopenia in older Brazilians was 17.0%. Sensitivity analysis showed rates of 20.0% in women and 12.0% in men [40]. In order to counteract the age-related muscle decline several interventions have been explored, including protein supplementation, testosterone replacement in men, estrogen replacement in women, growth hormone replacement, and treatment with vitamin D. To date, adequate protein intake and physical exercise are the most promising interventions aiming to prevent and/or delay the decline of muscle mass [41].

Oxidative stress and inflammation are implicated in the pathogenesis of sarcopenia [42]. It has been suggested that an increase of oxidative stress might activate apoptosis, leading to the loss of skeletal muscle fibers, thus contributing to the progression of sarcopenia [43]. Skeletal muscle tissue is unique, with respect to apoptosis, because muscle cells are one of the three cell types, along with osteoclasts and cytotrophoblasts, that are multinucleated [44]. The process by which nuclei are eliminated from multinucleated muscle fibers appears to be similar to apoptosis, since it involves chromatin condensation and DNA fragmentation. Moreover, it has been suggested that oxidative stress and the consequent mitochondrial genotoxic damage might play a causal role in the numerical loss of muscle fibers with aging [45].

Deregulation of redox homeostasis has emerged in recent years as a common pathogenetic mechanism and potential therapeutic target in collagen VI-related congenital muscular dystrophies, and in Duchenne muscular dystrophy, as well as in other more prevalent processes, such as age-related muscle loss [46]. Moreover, sarcopenia can be associated with obesity. This association is defined as sarcopenic obesity and represents a chronic condition, whose increase in prevalence has been related to parallel increases in the mean age of the population, the prevalence of obesity, and the changes in lifestyle during the last several decades [47]. In obese individuals, adipose tissue secretes both bioactive molecules, called “adipokines”, and ROS, which might represent the mechanistic link between obesity and its associated metabolic complications, including sarcopenia [48].

The aging of human skeletal muscle cells is marked by a progressive functional decline of mitochondria, resulting in the accumulation of ROS [43]. *Sod1^−^/^−^* mice lack the superoxide dismutase [Cu-Zn] 1 (CuZnSOD1) enzyme and undergo accelerated sarcopenia, i.e., display the characteristics of aging muscle in an accelerated manner. In *Sod1^−^/^−^* mice, muscle loss was accompanied by a progressive decline of mitochondrial function, with an increased mitochondrial generation of ROS and faster induction of mitochondrial-mediated apoptosis and loss of myonuclei. *Sod1^−^/^−^* mice also exhibited a strikingly increased number of dysfunctional mitochondria near neuromuscular junctions [49]. Increased ROS production is a necessary response to exercise of a sufficient intensity [50]. ROS and RNS (reactive nitrogen species, such as NO and the peroxynitrite anion) play important roles in the function of skeletal muscle, as they may mediate muscle adaptive responses, e.g., to physical exercise [51], by facilitating glucose uptake or inducing mitochondrial biogenesis [52,53]. However, during extended disuse periods, redox imbalance contributes to deleterious muscle remodeling via myonuclear apoptosis and atrophy, with the mediation of a redox-sensitive transcriptional regulator, NF-κB [54,55]. ROS can stimulate the production of TNF-α and IL-1β, through the activation of NF-κB-mediated pathways [56]. Systemic inflammation, impaired responses to stressors, and weakened regenerative capacity are all associated with sarcopenia. In Wistar rats, aging (40 weeks) was accompanied by reduced mitochondrial respiratory chain complex activities in saponin-skinned soleus muscle fibers, increased production of ROS and decreased transcription of the genes encoding mitochondrial superoxide dismutase 2 (*SOD2*), PPAR-γ coactivator-1β (*PGC-1β*) and sirtuin 1, in comparison with young (16 weeks) rats. Chronic intake of polyphenols normalized V_max_, decreased ROS production and enhanced *SOD2* and *PGC-1β* expression, in comparison with age-matched untreated rats [57].

Notably, sarcopenia is also accompanied by fat infiltration in the skeletal muscle, which can not only affect muscle quality and functional performance [58], but might render muscle tissue prone to oxidative stress-induced LPO. In disease processes marked by increased ROS production, novel 2,4-dinitrophenylhydrazine (DNPH)-reactive carbonyl groups in proteins, susceptible to spectrometric or antibody-mediated detection, might be either produced by the direct metal-catalyzed oxidation of aminoacyl side chains or introduced by stable adduct formation via the reaction of the latter with reducing sugars or reactive carbonyl species, including MDA and HNE. Age-related increases in protein carbonyl levels were detected in skeletal muscle by derivatization with DNPH, followed by fluorescent anti-DNPH antibodies, in descending ranking order, in the extracellular space, subsarcolemmal mitochondria, intermyofibrillar mitochondria and cytoplasm [59]. By the use of quantitative proteomics, a number of mitochondrial proteins susceptible to carbonylation in a muscle type-dependent (slow- vs. fast-twitch) and age-dependent manner were identified in Fischer 344 rat skeletal muscle. Fast-twitch muscle revealed twice as many carbonylated mitochondrial proteins than slow-twitch muscle, with 22 proteins showing significant changes (mostly increases) in carbonylation state with age. Ingenuity pathway analysis revealed that these proteins belonged to functional classes and pathways known to be impaired in muscle aging, including cellular function and maintenance, fatty acid metabolism and citric acid cycle. Although proof was not provided that carbonylation was responsible for any functional changes, these data delineated a catalog of protein targets deserving investigation, because of their potential implication in muscle aging [60]. In the gastrocnemius muscle of male C57B1/6 mice, a distinct increase of HNE adducts was noticed, upon the progression of age from 5 to 25 months. This was accompanied by increased expression of inducible nitric oxide synthase, decreased expression of G6PDH, activation of JNK, caspase 2, caspase 9, and inactivation of BCL-2, through phosphorylation at Ser70 [61]. Other observations have been collected in transgenic mice expressing a dominant-negative form of ALDH2 (ALDH2*2 Tg mice), which indicate a role of HNE as an inducer of apoptosis. Mice, in whose muscles ALDH activity was selectively attenuated, exhibited small body size, muscle atrophy, decreased fat content, osteopenia, and kyphosis, accompanied by increased muscular HNE levels [62].

Oxidative stress, in sarcopenic patients, can lead to increased LPO production. The mechanistic importance of apoptosis in the loss of muscle cells in age-related sarcopenia and the involvement of LPO-derived aldehydes in this process are supported by several studies. The abundance of protein carbonyl adducts was determined within skeletal muscle sarcoplasmic, myofibrillar, and mitochondrial protein subfractions from musculus *vastus lateralis* biopsies, using immunoblotting techniques, in two groups of 16 old males (“old” and “old sarcopenic”) [63]. Concentrations of cytoprotective proteins (e.g., heat shock proteins, αβ-crystallin) were also assayed. Aging was associated with increased mitochondrial (but not myofibrillar or sarcoplasmic) protein carbonyl adducts, independent of (stage-I) sarcopenia. Mitochondrial protein carbonyl abundance negatively correlated with muscle strength, but not muscle mass. According to this study, mitochondrial protein carbonylation increased moderately with age, and this increase might impact upon skeletal muscle function, but was not a hallmark of sarcopenia, per se. It should be considered, though, that the subjects under study were affected by low-grade (stage-I) sarcopenia [63].

A recent study, conducted with an urban Spanish cohort of elderly people (≥70 years), demonstrated a significant increase of MDA and HNE in blood samples of sarcopenic subjects, with respect to sarcopenia-exempt control subjects [64]. Notably, among several parameters examined in this study, only MDA and HNE levels were significantly associated with sarcopenia. These observations have been confirmed by studies demonstrating the presence of plasma MDA/HNE protein adducts in sarcopenic patients [65]. Interestingly, proteomic analysis of muscle extracts from adult and aged post-menopausal women demonstrated that ALDH2 and AKR1A1 were up-regulated in aged women, suggesting that the scavenging of reactive aldehyde products in skeletal muscle cells of the elderly was enhanced [66]. These results may be interpreted as a demonstration of the existence of a mechanism of adaptation of muscle cells to increased oxidative stress and to the consequently increased production of LPO-derived aldehydes.

Taken together, these results support a pathogenic link between oxidative stress, LPO-derived aldehydes, aldehyde-protein adducts and age-related sarcopenia in humans.

## 4. Immunosenescence

Immunosenescence takes a relevant part in the aging phenotype, and there is growing consensus on the idea that it might result from a progressive imbalance, favoring inflammatory over anti-inflammatory mechanisms, which some authors define as “inflammaging”. According to this view, continuing antigenic stimulation in the course of life, with accompanying oxidative stress, steadily leads, as enzymatic and non-enzymatic antioxidant defences fade off with age, to an up-regulation of inflammatory responses and a remodeling of immune responses, as revealed by the increased serum levels of pro-inflammatory cytokines, such as IL-6 and TNF-α, and a loss of efficacy of adaptive responses. Pro-inflammatory cytokines stimulate further the generation of ROS and cytotoxic LPO products.

As already well documented in other tissues and organ systems, accumulation of ROS and LPO products might cause significant damage to cell lipids, nucleic acids and crucial cell proteins also in cells of the immune system. This pro-inflammatory condition negatively affects the overall chances for good health, self-sufficiency and extended survival of the elderly and is a hallmark of the so-called “fragility” of the elderly [67,68]. Moreover, the remodeling of immunity strongly contributes to a number of age-associated diseases (infectious diseases, autoimmunity, cancer, metabolic, vascular and neurodegenerative diseases). Immunosenescence is best revealed by the changes in the modulation of survival of T cells, which become more resistant to damage-induced apoptosis. As a consequence, CD28-senescent T cells increase in number (with effector/memory cells, most of the CD8+ CD45RO+ CD25+ phenotype, prevailing over naïve cells), dysfunctional cells accumulate, and the immunological space in lymphoid tissues is reduced. Increased activation-induced cell death (AICD), in response to TNF-α, causes a progressive depletion of lymphoid niches, particularly of the naïve T cell compartment. At the same time, the generation of immunocompetent T cells in thymus declines with age [68,69,70,71,72]. The age-dependent shortening of DNA telomeres, which is associated with increased mortality in individuals over 65 years of age, contributes to the reduction of the potential for clonal expansion and differentiation of naïve T cells, which is revealed by the dramatic decay of T-cell-receptor excision circles (TREC). The overall outcome is a reduction in the repertoire of clonal antigenic specificities, which is reflected in a decreased ability to enact recall immune responses to formerly encountered antigens (e.g., cytomegalovirus, CMV) and to mount adaptive responses to novel antigenic stimuli, combined with an increased tendency to autoimmunity [68,69,70,71,72]. The inversion of the CD4+/CD8+ cell number ratio, the increased fraction of effector-memory cells and the seropositivity for CMV identify an immune risk phenotype in elderly patients [73]. Reduced B cell function with age reflects the decreases both of CD19+ cell number and of T helper-mediated cooperation with B cell responses. Age-associated changes in innate immunity include reductions of antigen uptake by DCs, phagocytosis and production of lymphocyte-derived chemotactic factor by macrophages, FCγRIII (CD16)-stimulated production of superoxide anion by neutrophils, and IFN-γ secretion and expression of activating NKp receptors by NK cells.

**Figure 2:**
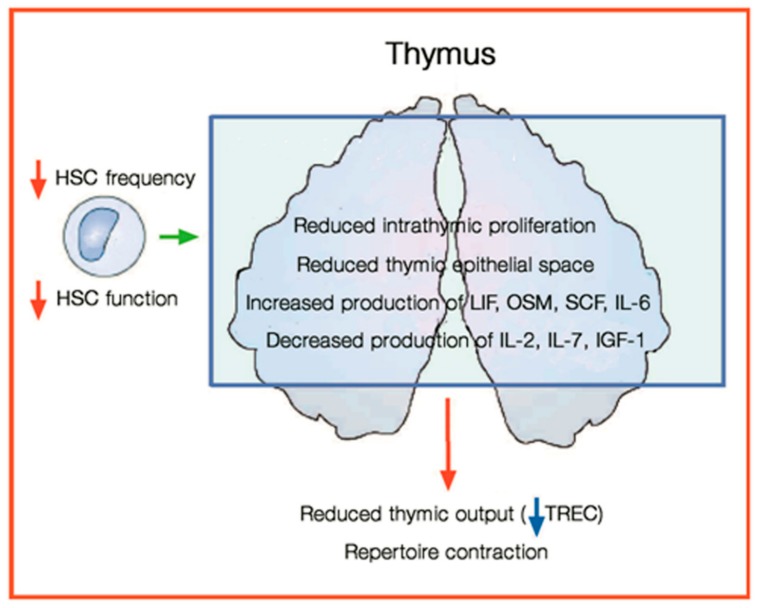
Main aspects of thymic involution. The decrease of hematopoietic cells causes a decrease of production of T cells from the thymus. HSC: hematopoietic stem cells; LIF: leukemia- inhibitory factor; OSM: Oncostatin M; SCF: stem cell factor; IL-2: interleukin 2; IL-6 interleukin 6; IL-7: interleukin 7; IGF-1: insulin-like growth factor 1; TREC: T cell receptor excision circles (modified from [68]).

Thymus involution is associated with a drastic reduction in organ volume and replacement of functional cortical and medullary areas with adipose tissue. This process, which starts early in life, is almost complete by the age of 40–50 years. Increasing formation of lipid-laden cells with aging has also been observed in lympho-hematopoietic organs, including the thymus [74,75]. Age-related thymic involution appears to reflect the compound effects of increased rates of thymocyte death in the thymus and decreased thymic differentiation and output of T cells. The former might result from intrathymic inflammation and lipotoxicity, the latter from the failure of stromal cell-dependent thymocyte maturation and survival (Figure 2) [76].

The levels of pro-inflammatory chemokines (MIP-1α, MIP-1β, RANTES) secreted by activated DC, macrophages and endothelial cells and recognized by CCR5 increase in the aging thymus, just like intrathymic lipid-laden multilocular and adipose cells (LLC), whose number is inversely related with thymic function [76]. It was suggested that LLC derive from CCR5-expressing, perithymic and perivascular preadipocytes, migrating into the aging thymus [77]. They produce pro-inflammatory cytokines (LIF, Oncostatin M, IL-6, TNF-α), thus creating a pro-oxidative, cytotoxic intrathymic *milieu*, which might favor thymocyte death [77]. The age-related alterations of lipid metabolism and redox balance were studied in mouse thymus and isolated thymocytes. An evaluation of the lipidomics profile of the whole thymus, between the ages of 4 weeks (young age) and 18 months (old age), revealed increased amounts of triacylglycerides, cholesterol, HNE, sulfatide ceramide and ganglioside GD1a in the aged thymus. Increased levels of cholesterol esters and HNE adducts were also found in isolated thymocytes from elder mice, compared with younger individuals. Increased levels of TNF-α and increased expression of CD204, a scavenger receptor of oxidized LDL, were detected in the thymic parenchyma of older individuals, as compared to younger ones. GH reduced thymic levels of TNF-α and HNE and increased the number of thymocytes, in accordance with several observations that indicated growth hormone as a powerful immunomodulating agent, stimulating thymopoiesis and limiting the number of adipocytes and fat locules in rodent and human thymus [74]. The levels of MDA and protein carbonyls (PC), as well as of oxidized and reduced glutathione and the activities of several antioxidant enzymes were measured in peripheral blood lymphocytes from 100 individuals, equally subdivided into groups of ages varying from 11–20 to 51–60 years. This study evidenced distinct, steady increases in MDA and PC levels with age, and decreasing glutathione levels and antioxidant enzyme activities, documenting a progressive redox imbalance with aging [78]. When rats subjected to ovariectomy, which undergo premature aging of the immune system, were subjected to dietary supplementation with polyphenolic antioxidants (soybean and green tea polyphenols), beneficial effects were observed on several parameters of the immune function (macrophage phagocytosis, chemotaxis and ROS production; lymphocyte mitogenic responses; NK activity) and redox balance (catalase and glutathione peroxidase activity, oxidized versus reduced glutathione ratio, MDA levels) of peritoneal leukocytes [60].

## 5. Myelodysplastic Syndromes

Myelodysplastic syndromes (MDS) include a heterogeneous range of stem cell disorders characterized by peripheral cytopenias and increased risk of progression to acute myeloid leukemia. The incidence of MDS increases markedly with age. It is conservatively estimated that >10,000 new cases of MDS occur in the United States annually [79]. In MDS, next-generation sequencing allowed the identification of molecular mutations in nearly 90% of the patients. Consequently, molecular mutation markers were integrated into current classification systems. The growing insights into molecular aspects of the pathogenesis of MDS may help to predict the possible evolution towards leukemia [80].

Most MDS patients have anemia and many develop transfusion dependence and iron overload (IOL). IOL is considered a negative independent prognostic factor, associated with a higher risk of leukemic transformation and shorter survival [81]. Serologic and molecular markers of oxidative stress have been evidenced in many patients with MDS [82], with the concentration of the LPO product MDA being significantly increased in patients with MDS and IOL, compared with IOL-exempt patients and control subjects. Moreover, both plasma nitrite and MDA concentrations were positively correlated with the ferritin levels, suggesting a relationship between IOL and the increased production of ROS in MDS [83]. In regard, it has to be noticed that the impact of iron on the redox balance of cells and body fluids is strictly dependent on transferrin saturation (TSAT). In the presence of normal TSAT, the M1 polarization of macrophages is decreased and the production of pro-inflammatory cytokines, ROS and LPO products are inhibited [84]. Conversely, patients with IOL and high TSAT show higher O_2_% saturation levels than patients at the time of diagnosis and normal controls. Antioxidant systems, with the exception of SOD, exhibit significant activity changes in IOL patients, compared with controls. Moreover, iron chelation treatment with deferoxamine has also been shown to reduce cytopenia in patients with MDS. In a study of 11 patients with MDS who were receiving deferoxamine for up to 60 months, increases in platelet and neutrophil counts were observed in 64% and 78% of patients, respectively [85]. Furthermore, mitochondrial dysfunction has been detected only in IOL cases, but not in control subjects [86]. High-level ROS production in MDS can be responsible for genotoxic damage to nuclear and mitochondrial DNA, resulting in genomic instability, which contributes to disease progression and leukemia onset [87,88].

Even though the role of ROS in MDS is well established, the role played by LPO products in the disease and its progression has not been completely assessed. It was suggested that the loss of ALDHs, which are mainly responsible for the metabolism of reactive aldehydes, might lead to the alteration of various cell processes, which may foster MDS progression to leukemic transformation [89]. Recent data indicate that ALDH1A1-defective cell lines, as well as primary leukemic cells, are sensitive to the treatment with drugs that directly or indirectly generate toxic ALDH substrates, including HNE. On the contrary, normal HSCs are relatively resistant to these compounds, suggesting that LPO-derived aldehydes could selectively kill ALDH1A1-defective leukemic cells [90]. According to these observations, in human lymphoid leukemic CDM-NKR cells, high concentrations of HNE caused significant cytotoxic effects on DNA synthesis and mitochondrial activity, whereas no significant toxicity of HNE was detected in normal hematopoietic precursor cells [91]. In addition, murine erythroleukemia (MEL) cells from HNE-treated mice exhibit a higher degree of differentiation, in comparison with dimethyl sulfoxide-treated MEL cells. These findings indicate that HNE, at concentrations physiologically detected in many normal tissues and in plasma, induces MEL cell differentiation, by modulating the expression of specific genes [92].

However, the role of HNE and other reactive aldehydes in the development of leukemia is controversial. The ALDH1A1 and ALDH3A1 isoforms are important for the metabolism of reactive aldehydes and ROS, and are expressed at high levels in HSCs. Indeed, the loss of these two isoforms resulted in a variety of effects on HSC biology, such as increased DNA damage and increased rates of leukemic transformation [93]. On the other hand, ALDH activity in human leukemic cells also mediates resistance to a number of drugs [94], and high levels of ALDH activity are predictors of poor therapeutic outcomes [95]. These conclusions are supported by the identification, by the in silico screening of the Gene Expression Omnibus database, using the *PTL* gene signature as a template, of 2 new agents, celastrol and HNE, which were able to eradicate acute myeloid leukemia (AML) at the bulk, progenitor, and stem cell level [96].

Serologic and molecular markers of oxidative stress in patients with MDS include increased concentrations of the LPO product MDA and the presence of oxidized bases in CD34+ cells. Potential mechanisms of oxidative stress include mitochondrial dysfunction via IOL and mitochondrial DNA mutation, systemic inflammation, and bone marrow stromal defects [82]. MDA levels in plasma correlated moderately with serum ferritin and free iron levels and were significantly higher in MDS patients with iron overload, when compared to healthy blood donors, once more emphasizing the role of oxidative stress in the development of MDS [97]. Moreover, a cytoprotective effect was reported of erythropoietin on the plasma membrane of erythroid cells in MDS, which was closely reminiscent of effects detected in certain conditions of impaired glucose metabolism, which were associated with increased LPO-dependent cell stress in the elderly [98]. The selective cytotoxic activities exhibited by LPO products towards transformed hematopoietic cells might be partly responsible for the effectiveness of hypomethylating drugs, widely employed in the chemotherapy of AML and MDS. In fact, the use of these chemotherapeutic agents is associated with the generation of enormous amounts of ROS. Antioxidant supplementation in these patients must be approached with caution, because of the high probability that it might result in significant reductions of the therapeutic efficacy of hypomethylating drugs, whose cytotoxic effect is probably mediated by plasma MDA concentrations, which increase significantly during the 14-day post-chemotherapy period [99].

In conclusion, it appears that the overall effects of LPO products within the context of MDS are strictly dependent on their concentrations, the degree of inflammation and the disease phase. Physiological HNE and MDA concentrations in an early phase of MDS, with low-grade inflammation and normal iron concentrations, seem to favor blast apoptosis and normal hematopoietic cell differentiation in bone marrow niches, whereas high HNE and MDA concentrations in later phases of MDS, with high-grade inflammation and higher than normal iron levels, might favor normal cell death and awry maturation of bone marrow cells [99].

## 6. Conclusions

From the overall body of evidence presented, the pathogenic involvement of oxidative stress and lipid peroxidation appears to be firmly grounded in all of the senile pathological and dysfunctional conditions examined in this review. In turn, increases in the production of ROS and reactive aldehydes from lipid peroxidation concur to induce apoptosis, which has been observed during aging in osteoblasts, muscle cells, thymocytes and hematopoietic cells. The use of antioxidants as an adjuvant therapy to counteract ROS increases in these disorders gave interesting results [26,87,100,101]. However, the efficacy of antioxidants in reducing the concentrations of aldehydes and/or protein-aldehyde adducts in blood or in tissues has not yet been measured. It is our conception that osteoporosis/osteopenia, sarcopenia, immunosenescence and myelodysplastic syndromes may all represent concurring expressions of the age-related decay of molecular turnover and repair capabilities in post-mitotic cells, altogether expressing themselves as the progressive multiorgan/multisystem failure of senescence. A few systematic redox proteomic approaches, pinpointing the modifications of distinct cell proteins with LPO products generated in the course of oxidative stress, have been conducted to date, mostly in the skeletal muscle of rodents. It is warranted that studies of this kind be extended to the other organ systems undergoing age-associated decay, and be complemented by functional studies as well. We anticipate that continuing investigation in this field may pave the way, in the end, to targeted therapeutic strategies aiming to alleviate the burden of morbidity and mortality associated with these disturbances.

## Abbreviations

ALDH	aldehyde dehydrogenase
AML	acute myeloid leukemia
apoB	apolipoprotein B
CMV	cytomegalovirus
DNPH	dinitrophenylhydrazine
HNE	4-hydroxynonenal
HSC	hematopoietic stem cell
IkB	inihibitor of κ light gene enhancer in B cells
IGF-1	insulin-like growth factor 1
IOL	iron overload
JNK	c-jun N-terminal kinase
LDL	low-density lipoprotein
LIF	leukemia-inhibitory factor
LLC	lipid-laden multilocular and adipose cell
LPO	lipid peroxidation
MAPK	mitogen-activated protein kinase
MDA	malondialdehyde
MDS	myelodysplastic syndromes
MEL	murine erythroleukemia
NK-κB	nuclear factor of κ light gene enhancer in B cells
OSM	oncostatin M
PC	protein carbonyl
PPAR-γ	peroxysome proliferator-activated receptor γ
SCF	stem cell factor
TNF-α	tumor necrosis factor alpha
TREC	T-cell receptor excision circle
TSAT	transferrin saturation
